# Ethno-pharmacological survey of herbal remedies used in the treatment of paediatric diseases in Buhunga parish, Rukungiri District, Uganda

**DOI:** 10.1186/s12906-019-2763-6

**Published:** 2019-12-05

**Authors:** Patience Tugume, Clement Nyakoojo

**Affiliations:** Department of Plant Sciences, Microbiology & Biotechnology, Makerere University, P.O Box 7062, Kampala, Uganda

**Keywords:** Traditional medicine, Herbal remedies, Children diseases, Buhunga, Digestive disorders

## Abstract

**Background:**

Plants have been used as a primary source of medicine since ancient times and about 80% of the world’s population use herbal medicine to treat different ailments. Plant use knowledge differs in space and time and thus requires documentation to avoid its loss from one generation to another.

**Methods:**

In order to accomplish the survey, semi-structured questionnaires were used. The data collected included names of plant species, parts used, ailments treated, growth habit, methods of preparation and mode of administration of the herbal remedies. Descriptive statistics were used to present the data in form of tables and a graph.

**Results:**

Results showed that 50 plant species belonging to 26 families were utilized in the treatment of paediatric diseases of which Asteraceae and Lamiaceae were the most common. Leaves (80%) were the most commonly used and decoctions were the main method of preparation. Twenty nine health conditions were treated out of which digestive disorders, malaria and respiratory tract infections were predominant. Herbs and shrubs were equally dominant.

**Conclusion:**

Herbal remedies are an important source of treatment for paediatric diseases in Buhunga Parish. However, there is need for collaboration between herbal medicine users and scientific institutions to help in the discovery of new drugs based on indigenous knowledge. Scientists ought to explore suitable methods of preparation and dosage formulations in order to achieve the best benefits from herbal remedies.

## Background

Plants have been used as a source of medicines for both humans and animals since time immemorial in crude forms such as decoctions, syrups, powders, infusions and ointments [1]. The use of herbal medicine in primary health care is still practiced in both developed and developing countries [2]. According to the World Health Organization (WHO) about 80% of the populations in developing countries use herbal medicine to meet their primary healthcare requirements [3, 4].

Traditional medicine is considered as a total sum of all practices, measures and procedures which have from time immemorial enabled Africans guard against disease. Kamatenesi [5] emphasises the importance of indigenous knowledge on plants species in the management of diseases. According to WHO [3], traditional medicine is characterized by a diversity of uses by people in different age groups. In Africa, reliance on folk medicine is partly due to the high cost of modern medicine and inaccessibility to health facilities [6].

Most of the ethnobotanical literature on traditional medicine is concentrated on the knowledge of traditional healers and largely overlooks domestic medicine and in particular the knowledge of women [7]. Domestic knowledge on the utilisation of herbal medicines needs to be prioritized in research in order to boost the interest of local people about their role in the management of diseases. The use of medicinal plant species in the treatment of children diseases is part and parcel of traditional knowledge that is handed down by word of mouth or orally hence documentation is necessary.

Over seven million children under five years of age die globally each year from preventable and treatable diseases especially pneumonia, diarrhoea and malaria [8]. Such children could be saved if interventions such as the use of antibiotics for pneumonia treatment, oral rehydration therapy for diarrhoea and use of insecticide-treated nets to prevent malaria are universally affordable [8].

In Uganda, the child mortality rate due to preventable diseases is still high at 90 deaths in 1000 live births [9]. Development of diseases in children is a particularly important concern because their health could be seriously threatened [10, 11]. In rural areas, owing to inadequate health facilities, high cost of conventional medicine, underfunding and mismanagement of the available health facilities, parents resort to the use of herbal medicine rather than modern medicine for treatment of children ailments [12, 13].

Although there is increased use of herbal medicine in Uganda, it could result into complications in case of improper prescriptions and dosages [3]. Much of the knowledge on the use of medicinal plants is possessed by traditional societies whose existence is threatened due to lack of systematic documentation. In addition to loss of indigenous knowledge, many medicinal plant species are facing extinction that could lead to genetic loss hence the need to document such information. The study aimed at documenting the medicinal plant species used in the treatment of paediatric diseases and their associated indigenous knowledge so that measures might be taken to develop new low cost therapies affordable by the rural poor.

## Materials and methods

### Study area

Buhunga Parish is located in Buhunga Sub-County, Rukungiri District in the Kigezi Sub-Region of Western Uganda. It is located between latitudes 0^o^45^’^18.7^″^S and longitudes 29^o^59^’^0.9.6^″^E. It is densely populated with a density of 328.8 people per square kilometre. The majority of the people are females. The parish consists of six villages namely Mutanoga, Kitookye, Rutooma, Kishaka, Nyamuyaga and Oruhita. The area is characterized by a hilly fertile landscape with occasional valleys that are dominated by livestock farms and banana plantations. There are two rainy seasons, one occurring from March to May and the other from September to November. The dominant tribes are the Banyakole and Bakiga.

## Methods

An ethnobotanical survey was conducted between December 2018 and February 2019 to document the therapeutic values of plant species used to treat childhood diseases, plant parts used, methods of preparation and administration. Standard ethnobotanical procedures [14] were followed. Data collection was based on structured interviews [15] and the responses were recorded by the researcher. The interviews were conducted in the local language of the participants since most of them were not formally educated. All interviews were conducted after obtaining verbal prior informed consent from participants. The first section of the questionnaire required demographic information of participants. The second section required local names of plant species used, diseases treated, modes of preparation and administration of the herbal recipes, plant part(s) frequently used, growth habits and habitats of the plant species.

The lottery method was used to randomly select 10 households from each of the six villages. Household heads were purposively selected for interview because they are the ones concerned about the healthcare of families. Plant specimens used in herbal recipes were collected with the aid of respondents and authenticated using their local names and standard text [16].Voucher specimens were collected, processed and deposited at the Makerere University Herbarium. Correctness of scientific names was checked in the database of International Plant Names Index (IPNI) https://www.ipni.org accessed on 1st February 2019.

## Results

### Demographic information of participants

The participants were mainly women (60%) above 18 years old. They had low education levels whereby 50% had not attained any formal education, 40% had attained at least primary education with the balance having attained secondary education level. A majority of participants were women because they are usually involved in caring for children. Most of the women were either single parents or widowed and thus with a heavy burden of providing for their children and had not attained any formal education compared to men who had at least attained primary and secondary levels of education. The low levels of education of participants imply limited employment opportunities and hence limited income. With the limited income, they have no option except the use of herbal medicine to provide healthcare for their children.

### Diversity of plant species used

The survey revealed a total of 50 medicinal plant species from 26 families were used to treat paediatric diseases. Scientific names, local names, family, growth form, disease treated, plant part(s) and mode of preparation and administration are presented in Table 1. Asteraceae was the most dominant family and contributed eight species followed by Lamiaceae (7), Fabaceae (5), Solanaceae (4) and Myrtaceae (3). The other families had one to two species each.

**Table 1:** Medicinal plant species, their habit, habitat, parts used, ailments treated, mode of preparation and administration

Family	Scientific name, Voucher No.	Local name (Runyankole/ Rukiga)	Habit	Habitat	Parts used	Ailment treated	Method of preparation & administration	Literature supporting traditional use of the plant species in other regions.
Acanthaceae	*Monechma subsessile* C. B. Clarke PT 040	Erazi	H	Along foot paths	L	-Stomachache -Epilepsy	-Decoction drunk	Not found
Aloeaceae	*Aloe vera* (L) Burm. f. PT046	Rukaka	S	Grasslands	L	-Stomachache	-Decoction drunk	Not found
Anarcadiaceae	*Mangifera indica* L.PT032	Omuyembe	T	Farmland	L B	-Cough	-Infusion drunk -Juice from macerated leaves drunk -Decoction drunk	-Oral wounds, tonsillitis and fever [17]
Apiaceae	*Centella asiatica* (L.) Urb. PT011		H	Swamps Farmland	Wp	-Dysentery -Constipation -Jaundice -Fever	-Infusion drunk -Juice from macerated leaves drunk	-Boils in Madagascar [18]
Asparagaceae	*Draceana fragrans* (L.) Ker Gawl. PT008	Omugorora	S	Bush Farmland	S, L, R	-Stomachache	-Decoction drunk	Leaves used for ear infections in Kibale [19]
Asteraceae	*Aspilia africana* (Pers) adama PT 006	Ekiterankuba	H	Bush Farmland	L	-Diarrhoea - Stomachache -Epilepsy	-Decoction drunk -Juice from macerated leaves drunk	-Child delay, wounds in Erute county [20]
	*Bidens pilosa* L. PT031	Enyabarashana	H	Bush Farmland	L R	-Fresh -wounds -Malaria -Worms -Constipation -Poisoning	-Leaves macerated and applied topically -Raw roots chewed -Decoction drunk	Also used for wounds in western Uganda [21] Stomachaches and jaundice in Peru [22]
	*Erlangea cordifolia* S. Moore PT001	Akatoma	S	Bush Abandoned lands	L	-Stomachache -Worms	-Decoction drunk	Stomach upsets in newly borns [19]
	*Erlangea tomentosa* S. Moore PT004	Ekyoganyanja	H	Bush Abandoned lands	L	-Stomachache -Jaundice	-Decoction drunk	Used for colic pains and stomachache in western Uganda [21]; Cough [19]
	*Vernonia amygdalina* Delile PT010	Omubirizi	S	Farmland Bush	L, R	-Worms -Malaria -Fever -Indigestion	-Decoction drunk	Burns, colic, malaria, syphilis, ulcers, wounds, skin rash [21]; Malaria, yellow fever [17]
	*Vernonia auriculifera* Hiern. PT019	Ekinyekanyeme	S	Bush	L	-Fever -Malaria -Stomachache	-Infusion drunk -Decoction drunk	Placenta removal [19]
	*Vernonia lasiopas* o. Hoffm PT025	Omujuma	S	Bush	L	-Worms	-Decoction drunk -Juice from macerated leaves drunk	Used against constipation, deworming, malaria, skin allergy, stomachache in Western Uganda [21] Malaria, stomachache [23]
	*Senecio ruwenzoriensis* S. Moore PT036	Omuzilafu	H	Grasslands Farmland	L	-Stomachache -Fever	-Decoction drunk -Decoction bathed	Not found
Bignoniaceae	*Markhamia lutea* K. Schum PT 018	Omusambya	T	Bush Farmland	R	-Diarrhoea	-Infusion drunk	Diarrhoea, gonorrhoea [19]
	*Spathodea companulata* P. Beauv.PT005	Munyara	T	Bush	L, B	-Allergy -Dysentery -Diarrhoea -Asthma	-Decoction drunk	Infertility, skin infections, Hernia [23]
Caricaceae	*Carica papaya* L. PT002	Omupapari	T	Farmland	L FR R	-Worms -Cough -Wounds	-Decoction drunk -Latex leaked -Macerated leaves	-Roots used against cough and diarrhoea in Ngai [24]
Chenopodiaceae	*Chenopodium ambrosioides* L. PT012	Musasizi	H	Bush	L	-Headache -Stomachache	-Macerated leaves rubbed in the head -Decoction drunk	For endoparasites; also as laxative in Peru [22]; Skin swellings in India [25]
	*Chenopodium opulifolium* Koch & Ziz PT020	Omwetango	H	Bush	L	-Malaria -Fever -Misfortune	-Infusion drunk -Decoction bathed -Macerated leaves mixed with water and child is bathed	Oral wounds, skin rash, toothache, sore throat [23]
Curcubitaceae	*Mormodica feotida* K. Schum PT 021	Akabombo	C	Farmland Hedges	L	-Malaria -Fever -Worms -Cough -Stomachache	-Decoction or infusion drunk	Yellow fever, cough and malaria in Iganga [17]
Euphorbiaceae	*Eurphobia hirta* L. PT014	Enkukuru	H	Farmland Compound	L WP	-Worms -Asthma -Wounds -Eye infections	-Decoction drunk -Leaves burnt and ash applied to wounds and boils	Sterility, diarrhoea, false teeth in Bulamogi [26]
	*Euphorbia turicalli* L. PT030	Oruyenje	S	Farmland Hedges	R Br	Stomachache -Sore throat -Snake bites	-Decoction drunk -Young branches roasted and chewed	Sap used to treat warts [23]
Fabaceae	*Albizia adianthifoli* (Schumach.) W. Wight PT043	Omushebeya	T	Farmland	L	-Fever	-Decoction drunk	Not found
	*Cassia occidentalis* Linn. PT 016	Omwitanjoka	S	Farmland Bush	L R	-Worms -Stomachache -Headache	-Infusion or decoction drunk	Paediatric cough [19]
	*Mimosa pudica* L. PT035	Kabarashaha	H	Farmland Home compounds	L, R	-Sores -Abcesses	-Macerated leaves/roots applied on affected part	Pre-mature ejaculation, measles [26]
	*Sesbania sesban* (L.) Merr PT 013	Omunyeganyeje	S	Farmland	L	-Stomachache -Abscesses	-Decoction drunk -Macerated leaves mixed with water and child bathed	High blood pressure, Diabetes [23]
Lamiaceae	*Iboza riparia* N.E. Br. PT 017	Omuravunga	H	Farmland	L	-Cough -Stomachache	-Decoction drunk	Not found
	*Leonotis nepetifolia* (L.) R. Br. PT 028	Ekicumucumu	H	Bush Along foot paths	L WP	-Stomachache -Dysentery -Convulsions -Malaria -Fever	-Decoction drunk -Decoction bathed	Colic, convulsions, deworming, dysentery, miscarriage, uterine pains in Western Uganda [21]
	*Ocimum kilimand* Schium PT 042	Obushonga	S	Farmland	L	-Headache -Stomachache	-Macerated leaves rubbed on the head -Decoction drunk	Not found
	*Ocimum gratissum* L. PT049	Omujaja	S	Bush	L	-Stomachache	-Decoction or infusion drunk	Not found
	*Ocimum suave* Willd. PT045	Esitimwa	S	Bush Farmland	L	-Stomachache	-Juice from macerated leaves drunk	Allergy, colic constipation, deworming, flue, stomachache, uterine pain, headache, splenomegaly [27]
	*Plectranthus barbatus Andr.*PT027	Rubatura	H	Farmland Along foot paths	L	-Stomachache	-Decoction drunk	Not found
	*Plecteanthus forskahlii Willd* PT023	Ekizera	H	Farmland	L	-Stomachache -Worms -Fever -Malaria	-Infusion or decoction drunk	Not found
Malvaceae	*Sida rhombiforia* L PT047	Orweza	H	Grasslands Farmland	L	-Misfortune	-Macerated leaves mixed with water and the child bathed	Not found
Melastomataceae	*Dissotis phaeotricha* (Hochst.) Hook. f. PT024	Mukurateitabye	H	Bush	L	-Malaria -Fever -Worms	-Decoction drunk	Not found
Meliaceae	*Azadirachta indica* A.Juss. PT007	Nimu	T	Farmland	L Fr S	-Worms -Malaria -Fever -Skin rash -Jaundice	-Decoction and infusion drunk -Oil from seeds drunk	Decoction used for skin rash in Ogun State Nigeria [28]; cough, fever [26]
Moraceae	*Ficus natalensis* Hochst. PT050	Ekitooma	T	Bush Farmland	B	-Influenza	-Decoction drunk	Not found
Myrtaceae	*Callistemon citrinus* (Curtis) Skeels PT009	Bottle brush (English)	T	Home compounds	L Br	-Cough -Common cold	-Decoction drunk -Mixed with leaves of *Eucalyptus grandis* and *Mangifera indica* boiled and inhaled	Cough [19]
	*Eucalyptus grandis* W. Hill PT003	Kalitusi	T	Farmland	L B	-Cough	-Leaves chewed -Infusion drunk -Decoction drunk	Not found
	*Psidium guajava* L. PT015	Omupera	T	Farmland	L B	-Cough	-Decoction drunk -Dried, pounded, powder added to cow ghee and leaked	Malaria, anaemia, fever, diarrhoea [17]
Passifloraceae	*Passiflora edulis* Sims PT027	Obutunda	C	Farmland	R T	-Stomachache	-Decoction drunk	Diarrhoea, Cough [17, 19]
Poaceae	*Cymbopogon citratus* Stapf PT039	Omutete	G	Grassland Farmland	L S	-Diarrhoea -Malaria -Fever -Ring worms	-Decoction or Infusion drunk -Seeds crushed mixed with water and drunk	Yellow fever [19]
	*Penissetum perpureum* Schum. PT037	Ekibingo	G	Grasslands Farmland	L R	-Blindness -Skin diseases	-Infusion used to wash the face -Infusion drunk	Heart disease [19]
Rubiaceae	*Pentas carnea* Benth. PT033	Ruhaya	S	Bush	L	-Stomachache	-Decoction drunk	Not found
	*Spermacoce princea* K. Schum PT041	Emarabyona	H	Along foot paths	Wp	-Stomachache -Worms	-Decoction drunk	Not found
Rutaceae	*Citrus sinensis* (L.) Osbeck PT022	Omuchungwa	T	Farmland	L	-Cough -Sore throat	-Leaves chewed -Mixed with other herbs, boiled and decoction drunk	Diarrhoea [17]
Solanaceae	*Datura innoxia* Mill PT026	Nyarweziringa	S	Swamps Farmland	L S Fr S	-Cough -Asthma -Skin diseases	-Infusion is drunk -Macerated leaves are applied on skin	Not found
	*Nicotiana tobacuum* L. PT029	Etaabe	H	Farmland	L	-Stomachache -Common cold -Influenza	-Leaves -Leaves are macerated and juice given to the child	Snake bites, Migraine [26]
	*Physalis peruviana* L. PT044	Ekituutu	C	Bush Along hedges	L	-Stomachache	-Decoction drunk	Hypertension, malaria, menstrual pains, nausea, splenomegaly,stomachache [21] Vomiting [26]
	*Solanum incanum* L. PT034	Entengotengo	S	Grasslands Farmland	Fr L R	-Wounds -Toothache -Prolapsed rectum	-Fruits are baked and used to massage the rectum -Juice from macerated leaves applied	Yellow fever [17]
Verbanaceae	*Lantana trifolia* L. PT038	Omuhukye	S	Bush Farmland	L	-Fresh wounds	-Infusion applied topically	Cough, amoebiasis [26];Sore throat, general malaise & gonorrhoea [29]

Key: Habit: H – Herb, S – Shrub, T – Tree, C – Climber, Parts used: L – Leaves, R- Roots, Fr – Fruits, S- Seeds, Wp – Whole plant, B – Bark,. Habitat means areas where respondents sourced the plant species; Farmland represents cultivated land, banana plantations

### Growth forms, habitat and plant parts used

Herbs formed the highest proportion of medicinal plant species (36%) closely followed by shrubs (32%), trees (22%), climbers (6%) and grasses (4%). The various plants parts used in herbal remedy preparation included fruits, leaves, roots, seeds, stems and the bark. In some cases whole plants were used. Leaves were the most frequently used part (58%), followed by roots (16%). The use of other plant parts was less common and in the range of 2–5%. However, there were instances where more than one plant parts of the same plant were used. For instance the leaves and roots of *Vernonia amygdalina* were used to treat malaria and worm infestation; leaves and stem bark of *Spathodea companulata* were used against allergy, dysentery, diarrhoea and asthma; seeds, leaves and roots of *Dracaena fragrans* were used to treat stomachaches while leaves and roots of *Mimosa pudica* were used against sores and abscesses.

For herbs, the whole plant was uprooted, leaves were plucked off from shrubs and for trees debarking was the main method of harvesting. Other methods of harvesting included digging out the roots, cutting young branches and picking seeds. Participants revealed that in instances where the species were rare, uprooting whole plants and digging out roots were avoided. They also reported controlled harvesting of plant parts in the range of 0.5–5.0 kg once or twice a week depending on the availability of the plant, severity of the ailment and age of the child.

Some plant parts were extracted from more than one habitat (Table 2). Farmlands were the most common sources of medicinal plant species with frequency of 47% followed by bushes 26%. Only two plant species *Datura innoxia* and *Centella asiatica* were extracted from swamps.

**Table 2:** Frequency of occurrence of medicinal plant species in different habitats

Habitat	Frequency	% Frequency
Farmland	34	47.2
Bush	19	26.3
Grasslands	6	8.3
Foot paths	4	5.6
Hedges	3	4.2
Home compounds	3	4.2
Abandoned lands	2	2.8
Swamps	1	1.4
Total	72	100

Figures are inclusive of each other

### Ailments treated using medicinal plant species

A total of 29 diseases were managed using medicinal plant species. The diseases were grouped into 10 categories of which digestive disorders, malaria and respiratory tract infections featured predominantly (Table 3). Species that treated the highest number of health conditions were; *Bidens pilosa*, *Mormodica foetida*, *Leonotis nepetifolia* and *Azadirachta indica* (Table 1). Some diseases were treated using a single plant species and in other cases a mixture of plant parts from different species were used. Monotherapy preparations were dominant compared to herbal concoctions. For instance jaundice was treated either using the roots of *Vernonia lasiopas* or leaves of *Erlangea tomentosa*; asthma using the whole plant of *Euphorbia hirta,* anal rectal prolapse using leaves of *Chenopodium murale,* constipation using leaves of *Vernonia amygdalina,* allergy using leaves of *Erlangea cordifolia,* epilepsy using leaves of *Sesbania sesban* and the root tuber of *Euphorbia tirucalli* as an antidote*.*

**Table 3:** Disease categories treated by different medicinal plant species

Disease category	Paediatric diseases	No. of plant species used (*N* = 50)	% of total species
Digestive disorders	Stomachaches, diarrhoea, constipation, dysentery, worm infestation	33	66
Malaria	Malaria	12	24
Respiratory tract infections	Sore throat, influenza, cough, common cold, asthma	11	22
Other infections	Ear, eye & oral infections	3	6
Skin infections	Wounds, ringworms, skin rash, allergy	9	18
Neurological disorders	Epilepsy, convulsions	3	6
Headaches		3	6
Inflammation	Abscesses, boils	1	2
Poisoning		1	2
Snake bites		1	2
Misfortune		1	2

Ailments that were managed using a combination of parts from different plants were; fever using a mixture of leaves of *Vernonia amygdalina* and *V. lasiopas* or leaves of *V. amygdalina and* roots of *Iboza riparia.* Cough was treated using a mixture of leaves of *Eucalyptus grandis, Mangifera indica* and *Callistemon citrinus.* A mixture of leaves of *V. amygdalina* and *Erlangea cordifolia* were used to treat headache. Dysentery was treated using leaves of *Chenopodium murale* and *V. amygdalina*, toothache using leaves of *V. lasiopas* and *Bidens pilosa.*

### Method of preparation and administration

**Fig. 1:**
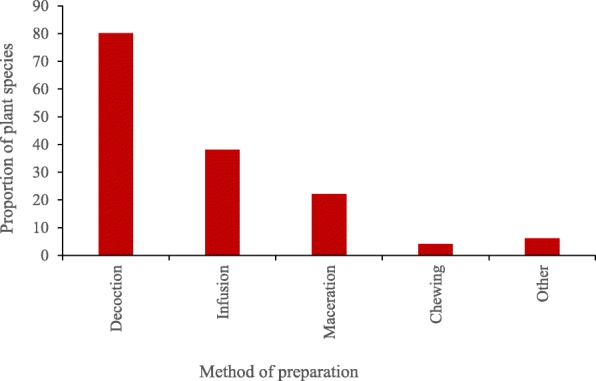
The figure illustrates the percentage of medicinal plant species used for making herbal remedies using different methods according to information obtained from respondents. The total number of species for calculation of percentages was 50. In some instances remedies from the same species could be prepared using more than one method. Water was the main ingredient used in preparation of herbal remedies in case decoctions and infusions. Method of preparation varied according to the plant species, parts used and sometimes conditions being treated

The major methods of preparation of plant extracts were decoction, infusion, maceration and chewing (Fig. 1). Water was the main solvent used in preparation of herbal therapies. Concoctions involved mixing water with an assortment of different plant parts. Decoctions were commonly used (80%) and were prepared by boiling plant materials in a specific quantity of water for 15–20 min and the mixture allowed to cool before administration. Decoctions were followed by infusions (38%) that involved pouring hot/warm water onto the plant material and allowing the mixture to cool. Maceration was used in 22% of the plant species. It involved crushing plant materials of a single species or a combination of more than one plant parts from different species to extract a liquid which was applied either topically or orally. Minor methods of preparation like pounding and roasting were used with low frequencies in the range of 2–6% of the medicinal plant species.

Some remedies were prepared by pounding the plant material and applying the paste topically on the affected part. In other instances the paste was mixed with water and the resulting mixture drunk. For children below three years of age, the leaves were chewed by the mother of the child who spat the resulting mixture into the child’s mouth. Children above three years of age chewed the leaves on their own. Some plant materials were roasted before applying them topically on the affected body parts. These were mainly used as poultices against abscesses, wounds and snake bites.

The modes of administration of herbal remedies included; leaking dry powdered plant materials especially leaves, drinking decoctions, infusions or concoctions, bathing the child with cold or warm extracts, applying crushed plant materials on the affected body parts, rubbing macerated plant materials on the affected part and mixing herbal preparations with food. Most respondents agreed that oral administration of herbal remedies was the most convenient for children.

There were similarities and variations in the use of plant species in the different villages in the study area. Some plant species were used in all the villages to treat the same ailments. For instance *Mangifera indica* and *Psidium guajava* for cough, *Vernonia amygdalina* against malaria, *Dracaena fragrans* and *Ocimum sauve* against stomach pains and *Albizia adianthifolia* against fever*.* In contrast, some plant species were used to treat different diseases in different villages. For instance *Spathodea campanulata* was used to manage asthma in Rutooma and Kishaka villages but used against dysentery in Mutanoga village. *Aspilia africana* was used to treat epilepsy in Oruhita village but used for diarrhoea in Kitookye and stomachache in Kishaka and Nyamuyaga villages.

## Discussion

Asteraceae, Lamiaceae and Solanaceae which were dominant families in the current study are among the most reported families with species used in herbal remedy preparation in Uganda [21, 23, 30, 31]. The high number of species from Asteraceae is attributed to the large number of its bioactive compounds [32]. Some of the plant species documented were used elsewhere in Uganda and other countries for treatment of either the same or different diseases (Table 1). The use of the same plant species to treat totally different conditions could be attributed to the fact that traditional knowledge is a closely guarded secret [33]. The use of same plant species for the same ailment in different localities indicates their cosmopolitan distribution and the fact that such plant species are effective for treatment of the specific ailments.

The high usage of herbs could be an indication of their abundance in the study area. These results are contrary to the findings of Kipkore et al. [34] and Katema et al. [35] where trees were dominant species for herbal medicine in Kenya and Ethiopia respectively.

The high frequency of occurrence of medicinal plant species in bushes is explained by the fact that plants that grow in the wild are rich in bioactive compounds [36]. Harvesting from farmlands and home gardens was prompted by the need to have medicinal plant species in the vicinity of homesteads to avoid travelling long distances in their search from the wild.

The use of leaves in herbal medicine preparation is common in other parts of Uganda [30, 37], Thailand [38] and Bolivia [32]. Leaves are mostly used because of their potency and fast regeneration ability. They are the main photosynthetic organs and also act as storage for exudates or photosynthates; some of which are of medicinal value [39, 40]. Their dominant use could also be attributed to the ease with which they are harvested. The bark and young branches were the least used parts because preparation of remedies from these parts is hectic and the perception by local people that they contain fewer bioactive ingredients compared to leaves and roots.

The large number of plant species used to manage digestive system disorders is a clear manifestation that these conditions were the most predominant in children. This is attributed to poor sanitation which encourages prevalence of pathogens [41]. The prevalence of malaria in the parish can be attributed to bushes and stagnant water in the proximity of the homesteads that provided breeding grounds for mosquitoes [42]. The majority of the households did not use insecticide treated mosquito nets due to lack of money thus perpetuating the problem of malaria [43].

The use of a mixture of plants in herbal medicine preparation has been shown to increase the effectiveness of herbal remedies due to synergistic effects [23]. Decoctions were the most common method of herbal medicine preparation. Boiling aids extraction of active ingredients from medicinal plant parts and preserves the herbal remedy longer than using cold extraction [31]. However, in some cases boiling may cause severe degradation of bioactive ingredients especially the aromatic compounds if it takes a long time [44]. Some studies have reported preparation of herbal remedies as decoctions [45]. Though the use of herbal medicine was embraced in the study area, the problem of lack of standardisation of dosages was eminent as also reported in another study [46].

## Conclusions

The people of Buhunga Parish have vast knowledge on medicinal plant species used in the treatment of various paediatric diseases. The medicinal plant species used are collected from a variety of habitats especially wild bush using several harvesting techniques some of which are destructive. The study creates awareness of the relevance of plant species in treatment of paediatric diseases, the therapeutic claims of which need to be assessed through phytochemical and pharmacological investigations to discover their bioactive ingredients. Further research focusing on preparation of herbal therapies with standardised dosages should be conducted in order to come up with recommended doses.

## Data Availability

All data generated and analysed during this study are included in the article.

## Abbreviations

IPNI: International Plant Names Index
WHO: World Health Organisation
